# Candesartan Reduces Neuronal Apoptosis Caused by Ischemic Stroke via Regulating the FFAR1/ITGA4 Pathway

**DOI:** 10.1155/2022/2356507

**Published:** 2022-09-07

**Authors:** Yubao Ding, Yue Lang, Hui Zhang, Yu Li, Xiao Liu, Minjie Li

**Affiliations:** ^1^Department of Neurology, The Third Hospital of Dalian Medical University, Dalian, Liaoning 116299, China; ^2^Department of Neurointensive Care Unit, The Second Hospital of Dalian Medical University, Dalian, Liaoning 116023, China; ^3^Major of Neurology, Dalian Medical University, Dalian, Liaoning 116044, China

## Abstract

Ischemic stroke (IS) is a general term for necrosis of brain tissue caused by stenosis, occlusion of arteries supplying blood to the brain (carotid artery and vertebral artery), and insufficient blood supply to the brain. Cerebral ischemia is the main kind of IS causing cell damage. However, the underlying mechanism still needs to be clarified further. In this study, it was demonstrated that FFAR1 was a hub gene in IS. The expression of FFAR1 was increased in PC12 cells with OGD/R treatment. FFAR1 deficiency inhibited cell viability and induced cell apoptosis, which was reversed by FFAR1 overexpression. Moreover, candesartan, as a compound targeting FFAR1, facilitated cell viability and reduced cell apoptosis. The expression of ITGA4 was also high in OGD/R-PC12 cells as FFAR1. Furthermore, FFAR1 deficiency retarded the increasing of cell viability and inhibition of cell apoptosis by downregulation of Bax and Cleaved Caspase-3 in OGD/R-PC12 cells with candesartan treatment. In conclusion, candesartan may regulate neuronal apoptosis through FFAR1/ITGA4 axis.

## 1. Introduction

In recent years, with the development of the economy and the improvement of living standards, stroke has become an important factor threatening health [1]. The main reason is cerebral ischemia, which subsequently causes damage to cell and apoptosis [2, 3]. At present, there are many studies on ischemic stroke, but its intrinsic molecular mechanism and treatment methods still need to be explored extensively. Cerebral ischemia causes cell apoptosis, which can lead to the irreversible death of neuronal cells [4]. Therefore, there is an urgent need for early prevention and effective treatment to reduce the occurrence and harm of stroke.

In recent years, more and more evidences have shown that FFA has multiple functions, including as an energy source and as a natural ligand for the group of orphan G protein-coupled receptors (GPCR) and free fatty acid receptors (FFAR), which participates in the interweaving of metabolism and immunity in many aspects, such as the regulation of inflammation and the secretion of peptide hormones. So far, several FFARs activated by FFAs with different chain lengths have been identified and characterized. In particular, FFAR1 (GPR40) and FFAR4 (GPR120) are activated by long-chain saturated fatty acids and unsaturated fatty acids. It is reported that the long-chain fatty acid receptor GPR40 has a significant inhibitory effect on poststroke central pain (one of the complications of cerebral ischemia and neuropathic pain syndrome) and has an antinociceptive effect. At the same time, some research has showed that it played an important role in astrocytes [5–7]. In addition, the overexpression of ITGA4 on MSCs enhanced transendothelial migration in vitro and improved the safety of intracarotid artery transplantation into stroke rats [8].

Candesartan, also known as candesartan cilexetil C8 intermediate, is an active metabolite derived from the hydrolyte of candesartan cilexetil. Candesartan is an antagonist of angiotensin II AT1 receptor. It antagonizes the vasoconstriction of angiotensin II by binding to vascular smooth muscle AT1 receptors, thereby reducing peripheral vascular resistance [9–11]. This drug is used to treat essential hypertension.

In this study, oxygen-glucose deficiency (OGD) was used to stimulate PC12 cells to establish *in vitro* cell models. We discussed the effects of candesartan and FFAR1/ITGA4 signal axis on PC12 cells with OGD.

## 2. Materials and Methods

### 2.1. Bioinformatics Analysis of GSE128623

Mice were divided into control group and ischemic injury groups at different time (4-42d). STEM (Short Time-series Expression Miner) cluster analysis [12] was used to investigate the relationship between gene expression and time. KEGG-GO analysis of gene clusters was done by R language (cluster Profiler, org.Hs.eg.db package). STRING (https://string-db.org/) was used to analyze protein-protein interaction networks (PPIs) and seek the interaction network of genes enriched in the process of neural ligand-receptor interaction relationship, which was graphed by Cytoscape 8.2 software.

### 2.2. Cells, Plasmids, and Antibodies

PC12 cells were treated with oxygen-glucose deprivation/reoxygenation to establish OGD/R model [13], and reoxygenation treatment was performed for different times (0 h, 24 h, and 48 h). siFFAR1 plasmids and FFAR1 overexpression plasmids were purchased from HanBio. The antibodies used in western blotting were listed as follows: antimouse Bcl-2 (1 : 1000, Santa, sc-73822, USA), antimouse Bax (1 : 1000, Santa, sc-20067, USA), antirabbit Cleaved Caspase-3 (1 : 1000, Abcam, ab2302, England), antirabbit FFAR1(1 : 1000, antibodies-online, ABIN3184898, Germany), antimouse ITGA4 (1 : 1000, Santa, sc-365209, USA), antimouse *β*-actin (1 : 1000, Santa, sc-8432, USA), and HRP-conjugated secondary antibodies to mouse (Jackson, 715-035-151) or rabbit (Jackson, 111-035-045). For candesartan treatment assay, after 24 hours of OGD modeling, PC12 cells were cultured with 1 *μ*M candesartan for 72 hours.

### 2.3. RNA Extraction and Real-Time qPCR

TRIzol reagent (Ambion, CA, USA) was used to isolate total RNA. After completing the extraction of RNA, reverse transcriptional kit was used to get cDNA, and RNA reverse transcription and RT-qPCR were performed as described as previously depicted [14]. cDNA was used as template to conduct the expression of FFAR1. The primers were listed as follows: FFAR1 forward, 5′-TCTCCTTCGGCCTCTATGTGG-3′; FFAR1 reverse, 5′-ACCAGGCTAGGGGTGAGAC-3′; GAPDH forward, 5′-AGGTCGGTGTGAACGGATTTG-3′; and GAPDH reverse, 5′-GGGGTCGTTGATGGCAACA-3′.

### 2.4. Western Blotting and Immunofluorescence

Protein was extracted using RIPA lysis buffer with proteasome inhibitor. Cells were digested with trypsin and washed with PBS and then spin down and treated with the lysis buffer. Then, supernatant was collected by centrifugation and boiled at 95°C for 5 min. Next, proteins were added into gel for electrophoresis, which was then transferred onto PVDF membrane and immunoblotted and visualized with chemiluminescent ECL reagent. The same membrane was stripped for reuse for other antibody incubations. For immunofluorescence assay, cerebral cortex tissue was collected, then sliced, fixed, and incubated with FFAR1 and NeuN.

### 2.5. CCK8 Assay

PC12 cells were treated with reoxygenation for different time, and 5000 cells were seeded into 96-well plates, which were treated with candesartan and transfected with the indicated plasmids. For the indicated period of cell growth, cell lines were treated with CCK8 reagent and then tested by microplate reader. All procedures were conducted according to the manufacturer's instruction.

### 2.6. Cell Apoptosis Analysis

PC12 cells were treated with the same plasmids and/or reagent, cells were spin down and collected, counted, and then stained by PI and/or annexin V following manufacturer's instruction. Finally, flow cytometry was used to analyze cell apoptosis.

### 2.7. Prediction and Functional Analysis of Target Drugs

The structure was predicted by PubChem (https://pubchem.ncbi.nlm.nih.gov). Then, the online software SwissTargetPrediction (http://www.swisstargetprediction.ch/) was used to analyze the targets of candesartan. Functional enrichment was analyzed by Metascape (https://metascape.org/gp/index.html#/main/step1) and Enrichr (https://maayanlab.cloud/Enrichr/), respectively.

### 2.8. Statistical Analysis

Student's *t*-test and one-way ANOVA were used for statistical analysis. Data was presented as mean ± SEM of three independent experiments. *P* < 0.05 was considered to be significant.

## 3. Results

### 3.1. STEM Cluster Analysis of Mainstream Gene Expression Trends and Functional Analysis before and after Cortical Stroke

**Figure 1:**
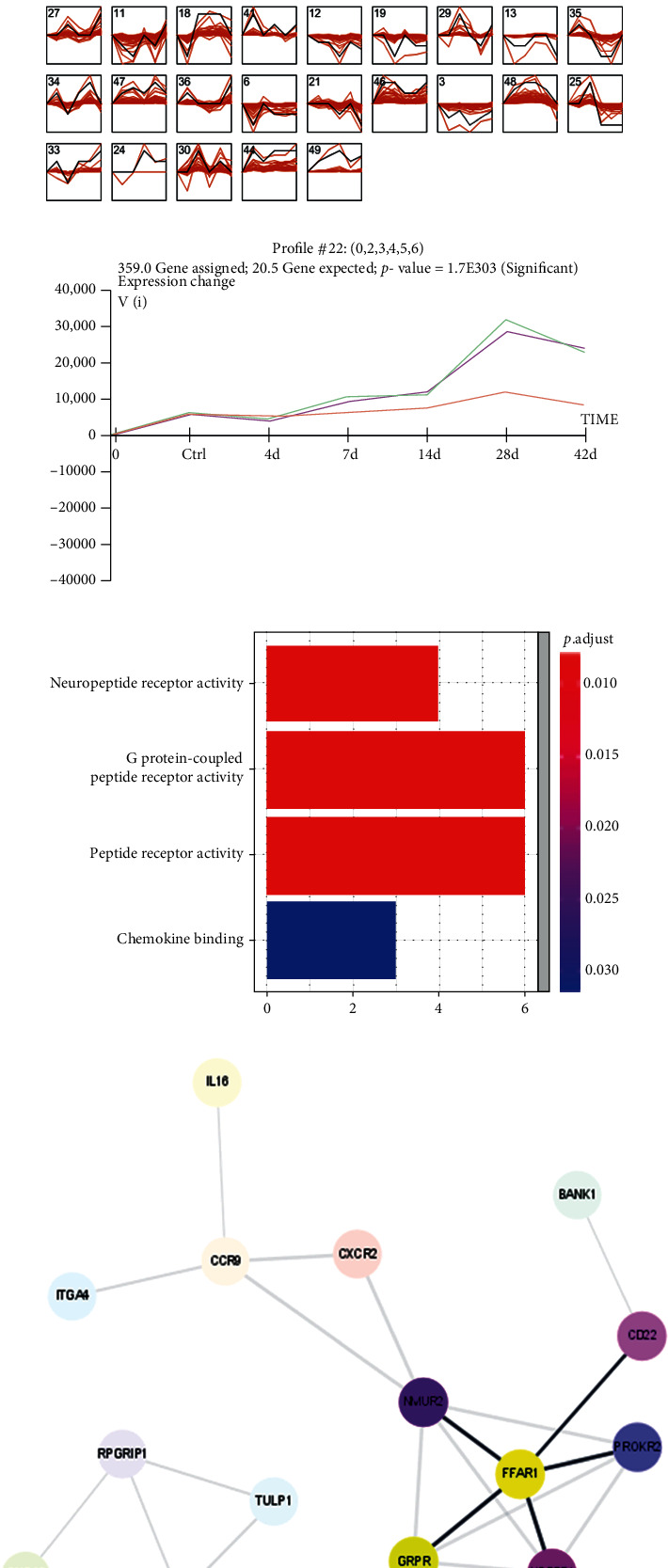
STEM cluster analysis of mainstream gene expression trends and functional analysis before and after cortical stroke in GSE128623. (a) Cluster analyzed gene expression before and after cortical stroke in GSE128623 (cluster order: gene number; profile order: significance). (b) The correlation between gene expression and time in profile 22 (359 genes). (c) KEGG-GO analysis of different expression genes. (d) PPI analysis of the DEGs in profile 22 by STRING (https://string-db.org/).

Firstly, in order to clarify the underline relationship between gene expression and time course, we used Short Time-series Expression Miner cluster to analyze the change of mainstream gene expression trends in GSE128623 (mice in ischemic injury model). As shown in Figure 1(a), the change of gene expression trends was significant in six profiles, including profiles 15, 22, 37, 45, 28, and 23. However, only the expression of profile 22 gene cluster was upregulated over time. Gene expression showed an upward trend in profile 22 (number of genes: 359), indicating that the expression of genes in profile 22 was upregulated with the increase of the time of brain injury in mice (Figure 1(b)). Therefore, the gene clusters in profile 22 were further analyzed by KEGG-GO, and the most significant and abundant pathway associated with neuromodulation for these genes was neural ligand-receptor interactions (Figure 1(c)). Next, STRING was used to analyze the protein-protein interaction networks and search the hub gene in profile 22. As shown in Figure 1(d), FFAR1 was a hub gene located at the core of the PPI network diagram.

### 3.2. The Expression of FFAR1 Was Upregulated in the OGD/R Model of Cells In Vitro

**Figure 2:**
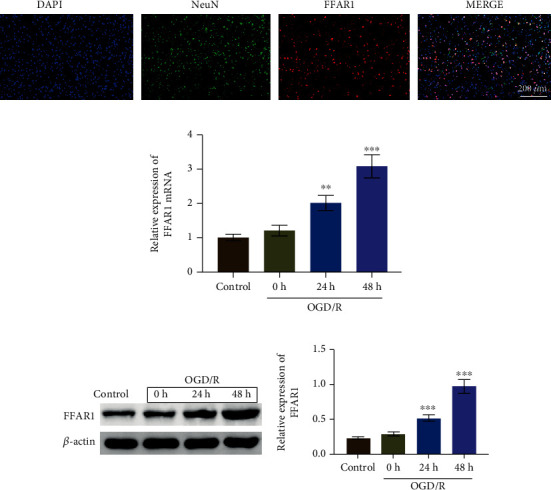
The expression of FFAR1 was upregulated in the OGD/R model of cells in vitro. (a) Immunofluorescence was used to measure the colocalization of FFAR1 and neurons. (b) PC12 cells were treated with oxygen-glucose deprivation/reoxygenation for 0 h, 24 h, and 48 h; the expression of FFAR1 was measured by RT-qPCR. (c) Western blotting assay was used to analyze FFAR1 expression in PC12 cells with OGD/R treatment. Data was expressed as mean ± SD from three independent experiments. ^∗∗^*P* < 0.01 and ^∗∗∗^*P* < 0.001.

Based on the core location of FFAR1 in network of profile 22, we analyzed the expression of FFAR1 in OGD/R model of PC12 cells. First of all, we used immunofluorescence to measure the colocalization of FFAR1 and NeuN, as shown in Figure 2(a), FFAR1 was expressed in neuron, and FFAR1 and NeuN were colocalized in neuron cells. Next, we further measured the expression of FFAR1 by RT-qPCR; as shown in Figure 2(b), FFAR1 mRNA expression was significantly upregulated in OGD/R model with reoxygenation treatment for 24 h and 48 h and the expression of FFAR1 was increased over time. Apart from this, the similar expression trends of FFAR1 at the protein level was observed (Figure 2(c)). Taken together, our data indicated that the expression of FFAR1 is high in the OGD/R model.

### 3.3. FFAR1 Regulated the Survival and Apoptosis of OGD/R-PC12 Cells

**Figure 3:**
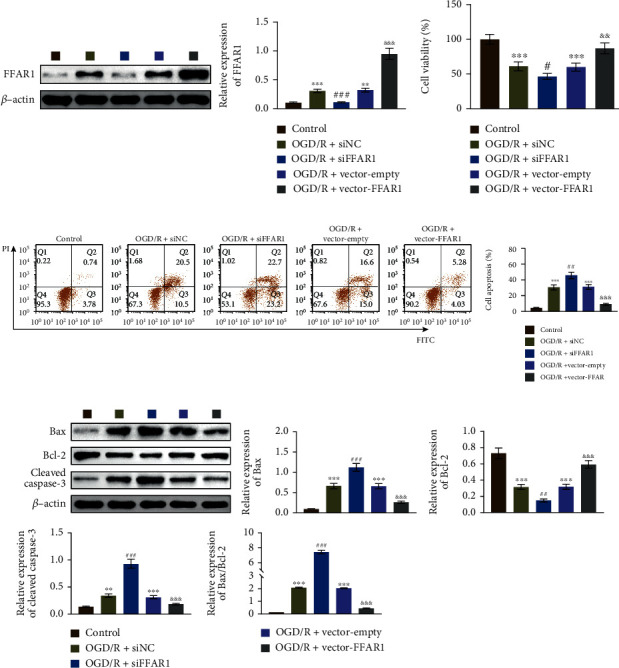
FFAR1 induced the survival of OGD/R-PC12 cells. (a) Construction of OGD/R model of PC12 cells through treating cells with reoxygenation for 48 h. The cells were also transfected with the indicated plasmids, and the expression of FFAR1 was measured by western blotting. (b) The cell viability was analyzed by CCK8 assay in FFAR1 deficiency or overexpressed PC12 cells with OGD/R treatment for 48h. (c) Flow cytometry analyzed cell apoptosis in FFAR1 deficiency or overexpressed PC12 cells with OGD/R treatment for 48 h. (d) Apoptosis-related proteins were analyzed by western blotting. Data was expressed as mean ± SD from three independent experiments. ^∗∗^*P* < 0.01, ^∗∗∗^*P* < 0.001, ^#^*P* < 0.05, ^##^*P* < 0.01, ^###^*P* < 0.001, ^&&&^*P* < 0.001, ^∗^compared to a mock group, ^#^compared with the siNC group, and ^&^compared with empty group.

Next, we transfected the siFFAR1 and FFAR1 overexpression plasmids into OGD/R-PC12 cells. As shown in Figure 3(a), the expression of FFAR1 was decreased in OGD/R-PC12 cells transfected with siFFAR1, which was upregulated in OGD/R-PC12 cells transfected with FFAR1 plasmid. To clarify the function of FFAR1 in OGD/R-PC12 cells, CCK8 was used for measuring cell viability. As shown in Figure 3(b), cell viability was inhibited in OGD/R-PC12 cells and further constrained by siFFAR1 transfection. However, the overexpression of FFAR1 retarded the suppression of cell viability in OGD/R-PC12 cells. Moreover, we also analyzed the function of FFAR1 in cell apoptosis by flow cytometry. As shown in Figure 3(c), cell apoptosis was induced in OGD/R-PC12 cells and further increased in OGD/R-PC12 cells with siFFAR1 transfection. However, overexpression of FFAR1 antagonized the facilitation of cell apoptosis in OGD/R-PC12 cells. Mechanically, the expression of apoptosis-related proteins was analyzed in OGD/R-PC12 cells. FFAR1 deficiency promoted the expression of Bax and Cleaved Caspase-3, while inhibiting Bcl-2 expression in OGD/R-PC12 cells. In addition, overexpression of FFAR1 increased Bcl-2 expression and the expression of Bax and Cleaved Caspase-3 in OGD/R-PC12 cells (Figure 3(d)). Collectively, our results indicated that FFAR1 positively regulated cell viability and negatively regulated cell apoptosis.

### 3.4. Compound-Genomics Target Drug Prediction and Functional Analysis

**Figure 4:**
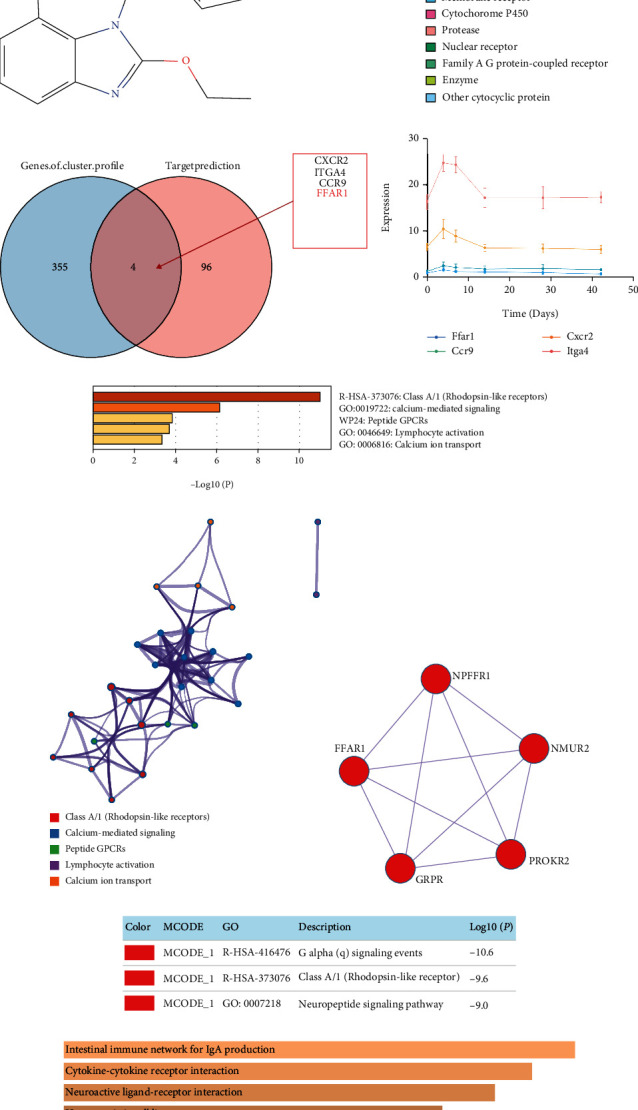
Compound-genomics target drug prediction and functional analysis. (a) Prediction of compound targeting FFAR1 was analyzed by PubChem (https://pubchem.ncbi.nlm.nih.gov). (b) Target prediction of candesartan was analyzed by SwissTargetPrediction (http://www.swisstargetprediction.ch/), and the top 25 targets were classified to obtain the target class pie chart. (c) Then, Venn analysis (http://bioinformatics.psb.ugent.be/webtools/Venn/) of the compound targets (100 genes) with profile 22 (359 genes) was performed. (d) The correlation between gene expression and time of the intersection genes was analyzed by Venn analysis. (e) Bar graph of enriched terms across input gene lists by Metascape (https://metascape.org/gp/index.html#/main/step1). (f) Network of enriched terms. (g, h) Protein-protein interaction network and MCODE components were identified in the gene lists. (i) Enrichment analysis of the input gene was listed by Enrichr (https://maayanlab.cloud/Enrichr/).

We further used PubChem to predict the compound targeting FFAR1. As shown in Figures 4(a) and 4(b), we acquired the structure of candesartan and predicted its targets by SwissTargetPrediction. There were 40% targets which were associated with family A G protein-coupled receptors. Moreover, only 4 intersection genes (CXCR2, ITGA4, CCR9, and FFAR1) were included both in the targets of candesartan (100 genes) and profile 22 (359 genes) (Figure 4(c)). Similarly, we also analyzed the correlation of the gene expression with time course. As shown in Figure 4(d), the expression of FFAR1 was almost constant over time. Furthermore, the input genes were analyzed by Metascape and Enrichr for enrichment analysis, and these genes were closely involved in neuroregulation (Figures 4(e)–4(i)). Collectively, our data indicated that candesartan may participate in poststroke recovery by targeting FFAR1.

### 3.5. Candesartan Regulated the Survival and Apoptosis of OGD/R-PC12 Cells

**Figure 5:**
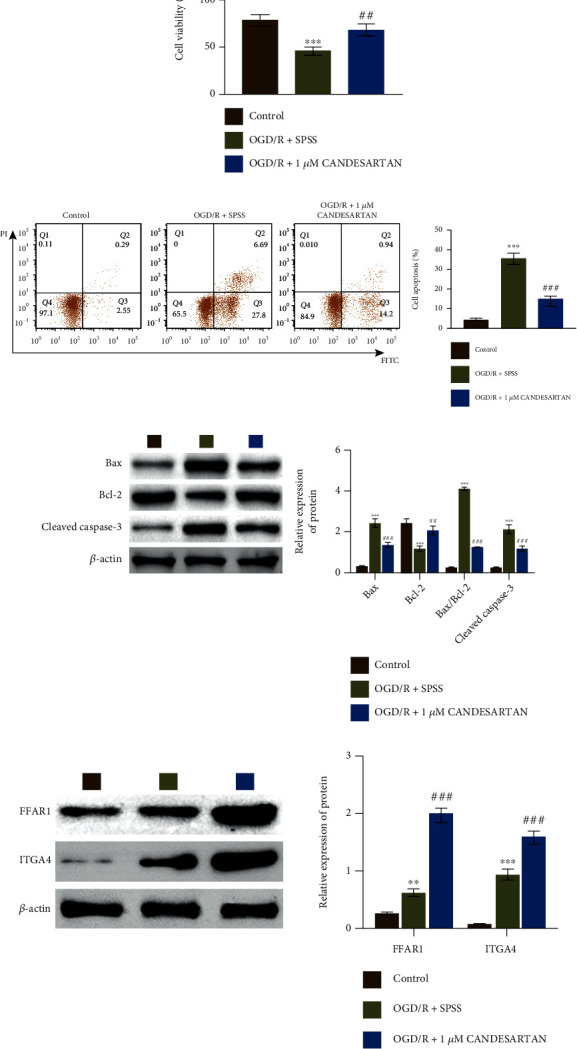
Candesartan increased the survival of OGD/R-PC12 cells. (a) OGD/R model PC12 cells were divided into three groups: control, OGD/R + SPSS, and OGD/R + 1*μ*M candesartan; then, cell viability was analyzed by CCK8. (b) Flow cytometry was used to analyze cell apoptosis in OGD/R model of PC12 cells with candesartan treatment. (c, d) Western blotting assay was used to measure the expression of Bax, Bcl-2, Cleaved Caspase-3, FFAR1, and ITGA4. Data was expressed as mean ± SD from three independent experiments. ^∗∗^*P* < 0.01, ^∗∗∗^*P* < 0.001, ^##^*P* < 0.01, ^###^*P* < 0.001, ^∗^compared to control group, and ^#^compared with the OGD/R+SPSS group.

To elucidate the function of candesartan, we also analyzed the effect of candesartan on cell viability and apoptosis in OGD/R-PC12 cells. As shown in Figure 5(a), candesartan treatment increased cell viability in OGD/R-PC12 cells. However, candesartan reduced cell apoptosis in OGD/R-PC12 cells (Figure 5(b)). Additionally, the upregulation of Bax and Cleaved Caspase-3 was restrained in OGD/R-PC12 cells with candesartan treatment, whereas the prohibition of Bcl-2 was released by candesartan in OGD/R-PC12 cells (Figure 5(c)). Interestingly, the expression of both FFAR1 and ITGA4 was increased in OGD/R-PC12 cells with candesartan treatment (Figure 5(d)). Taken together, our data suggested that candesartan positively regulated cell survival in OGD/R-PC12 cells.

### 3.6. Candesartan Regulated the Survival and Apoptosis of OGD/R-PC12 Cells by Targeting the FFAR1/ITGA4 Signal Axis

**Figure 6:**
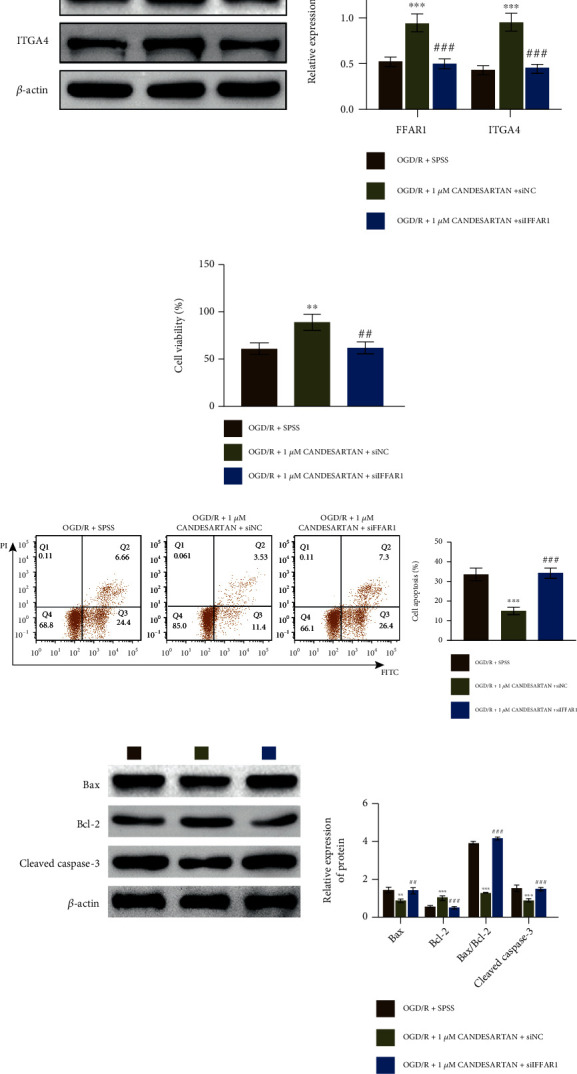
Candesartan regulated the survival and apoptosis of OGD/R-PC12 cells via targeting the FFAR1/ITGA4 signal axis. (a) OGD/R model of PC12 cells were divided into three groups: OGD/R + SPSS, OGD/R + 1*μ*M candesartan + siNC, and OGD/R + 1*μ*M candesartan + siFFAR1; then, the expression of FFAR1 and ITGA4 was measured by western blotting. (b) CCK8 assay was used to analyze cell viability in the three group of PC12 cells. (c) Flow cytometry was used to analyze cell apoptosis in OGD/R model PC12 cells with candesartan and/or siFFAR1 treatment. (d) The expression of Bax, Bcl-2, and Cleaved Caspase-3 in PC12 cells with candesartan and/or siFFAR1 treatment was measured by western blotting. Data was expressed as mean ± SD from three independent experiments. ^∗∗^*P* < 0.01, ^∗∗∗^*P* < 0.001, ^##^*P* < 0.01, ^###^*P* < 0.001, ^∗^compared to a mock group, ^#^compared with the siNC group, and ^&^compared with empty group.

To further investigate the function of FFAR1 in candesartan-treated OGD/R-PC12 cells, we examined the expression of ITGA4 in candesartan- and siFFAR1-treated OGD/R-PC12 cells. As shown in Figure 6(a), the expression of ITGA4 was downregulated in OGD/R-PC12 cells with candesartan and siFFAR1 treatment. Functionally, the increasing of cell viability was reduced in OGD/R-PC12 cells with candesartan and siFFAR1 treatment (Figure 6(b)). FFAR1 deficiency antagonized the suppression of cell apoptosis in OGD/R-PC12 cells with candesartan treatment (Figure 6(c)). Furthermore, the decreasing of Bax and Cleaved Caspase-3 was retarded in OGD/R-PC12 cells with candesartan and siFFAR1 treatment, while Bcl-2 expression was inhibited in OGD/R-PC12 cells with candesartan and siFFAR1 treatment (Figure 6(d)).

**Figure 7:**
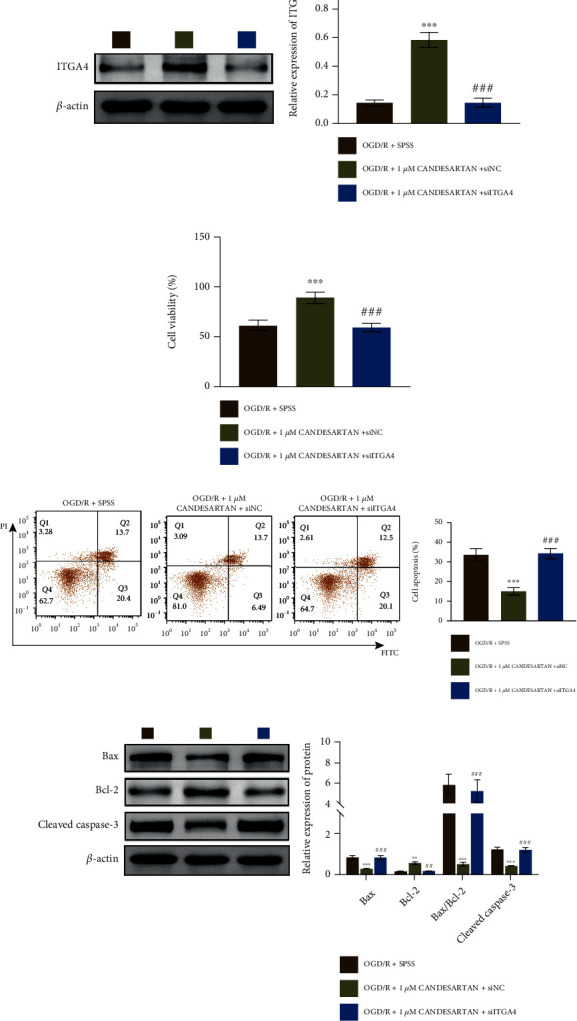
The survival and apoptosis of OGD/R-PC12 cells was regulated by FFAR1/ITGA4 signal axis. (a) OGD/R model PC12 cells were divided into three groups: OGD/R + SPSS, OGD/R + 1*μ*M candesartan + siNC, and OGD/R + 1*μ*M candesartan + siITGA4; then, the expression of FFAR1 and ITGA4 was measured by western blotting. (b) CCK8 assay was used to analyze cell viability in the three group of PC12 cells as in (a). (c) Flow cytometry was used to analyze cell apoptosis in OGD/R model of PC12 cells with candesartan and/or siFFAR1 treatment. (d) The expression of Bax, Bcl-2, and Cleaved Caspase-3 in PC12 cells with candesartan and/or siITGA4 treatment was measured by western blotting. Data was expressed as mean ± SD from three independent experiments. ^∗∗^*P* < 0.01, ^∗∗∗^*P* < 0.001, ^##^*P* < 0.01, ^###^*P* < 0.001, ^∗^compared to a mock group, ^#^compared with the siNC group, and ^&^compared with empty group.

In addition, we also studied the function of ITGA4 in OGD/R-PC12 cells with candesartan and/or siITGA4 treatment. We measured the expression of ITGA4 in OGD/R-PC12 cells with candesartan and siITGA4 treatment. As shown in Figure 7(a), the expression of ITGA4 was decreased in OGD/R-PC12 cells with candesartan and siITGA4 treatment compared to single treatment of candesartan. Functionally, the increasing of cell viability was also inhibited in OGD/R-PC12 cells by candesartan and siITGA4 treatment (Figure 7(b)). ITGA4 deficiency released the suppression of cell apoptosis in OGD/R-PC12 cells with candesartan treatment (Figure 7(c)). Furthermore, the decreasing of Bax and Cleaved Caspase-3 was relieved in OGD/R-PC12 cells with candesartan and siITGA4 treatment, while Bcl-2 expression was inhibited in OGD/R-PC12 cells with candesartan and siITGA4 treatment (Figure 7(d)).

Collectively, our data indicated that candesartan regulated the survival and apoptosis of OGD/R-PC12 cells through targeting the FFAR1/ITGA4 signal axis.

## 4. Discussion

As a heterochromatic cell line derived from rat adrenal medulla, PC12 cells are commonly used in research on function of nerve cell line, which belongs to a tumor of the sympathetic nervous system. Here, we used oxygen-glucose deficiency (OGD) to stimulate PC12 cells to establish cell models in vitro. Firstly, we demonstrated that FFAR1 was a hub gene in ischemic stroke by STEM cluster analysis, KEGG-GO enrichment analysis, and PPI analysis from GSE128623. Then, we confirmed that the expression of FFAR1 was upregulated in PC12 cells with OGD/R treatment. Secondly, FFAR1 positively regulated cell viability and negatively mediated cell apoptosis via facilitating the expression of Bcl-2 and inhibiting the expression of Bax and Cleaved Caspase-3 in PC12 cells with OGD/R treatment. Thirdly, the online prediction result revealed that FFAR1 was a target of candesartan, and candesartan exerted the same function in cell proliferation, cell apoptosis, and related protein expression as FFAR1. Interestingly, the expression of FFAR1 and ITGA4 was increased in OGD/R-PC12 cells with candesartan treatment. Additionally, the increasing of cell viability and inhibition of cell apoptosis were antagonized by siFFAR treatment in OGD/R-PC12 cells with candesartan treatment.

Central poststroke pain (CPSP) is one of the complications of cerebral ischemia and neuropathic pain syndrome, which is related to specific somatosensory abnormalities. Previous study reported that GPR40, also known as FFAR1, was a long-chain fatty acid receptor and had an antinoxious effect in CPSP [6]. GPR40, as the first identified G protein-coupled receptor, delivered nonenzyme-generated nitrooxidation products, particularly TAAs, and was involved in HI encephalopathy [7]. The activation of GPR40 attenuated the neuroinflammation induced by GMH through activating the PAK4/CREB/KDM6B signaling pathway [15]. DHA significantly induced growth inhibition and apoptosis of human androgen-dependent prostate cancer cells via inactivating YAP by promoting phosphorylation and cytoplasmic translocation pathways (FFAR1/FFAR4-Gas-PKA-Hippo) [16]. We also demonstrated that FFAR1 induced cell survival in OGD/R-PC12 cells.

As an angiotensin II receptor agonist, candesartan is widely used in cardiovascular disease, metabolic syndrome, and brain injury. In animal models of stroke, systemic administration of candesartan can effectively protect cerebral blood flow and reduce blood-brain barrier leakage, cerebral hemorrhage, neuroinflammation and neuronal damage [17, 18], traumatic brain injury [19, 20], and Alzheimer's disease [21]. In the model of stroke-prone spontaneous hypertension rats (SHRSPs), the use of candesartan can reduce the incidence of stroke [22, 23]. Candesartan can reduce the infarct size, improve signal transduction, increase cerebral blood flow, and stimulate the neurotrophic factor BNDF/TrkB system in the rat MCAO model [24, 25]. Clinical studies on the prevention and treatment of stroke with candesartan have also been carried out. The efficacy of candesartan in the prevention of stroke was confirmed in the Cognitive and Prognostic Study of the Elderly (SCOPE) [26]. Previous study demonstrated that candesartan participated in the regulation of VSMC proliferation via regulating miR-301b/STAT3 cascades [27]. Here, we also demonstrated that candesartan positively regulated cell proliferation via FFAR1/ITGA4 axis in OGD/R-PC12 cells. However, the underlying molecular mechanisms still need to be investigated.

In conclusion, candesartan reduced the apoptosis of OGD/R-PC12 cells through regulating FFAR1/ITGA4 signal axis. FFAR1 may be a key target of candesartan for treating ischemia stroke. However, how candesartan regulates FFAR1/ITGA4 axis in the treatment of ischemic stroke needs further investigations.

## Data Availability

All data generated or analyzed during this study are included in this published article.
